# Direct observation of high spin polarization in Co_2_FeAl thin films

**DOI:** 10.1038/s41598-018-26285-9

**Published:** 2018-05-23

**Authors:** Xiaoqian Zhang, Huanfeng Xu, Bolin Lai, Qiangsheng Lu, Xianyang Lu, Yequan Chen, Wei Niu, Chenyi Gu, Wenqing Liu, Xuefeng Wang, Chang Liu, Yuefeng Nie, Liang He, Yongbing Xu

**Affiliations:** 1 Jiangsu Provincial Key Laboratory of Advanced Photonic and Electronic Materials, Collaborative Innovation Center of Advanced Microstructures, School of Electronic Science and Engineering, Nanjing University, Nanjing, 210093 China; 2 Department of Physics, Southern University of Science and Technology, Shenzhen, Guangdong, 518055 China; 3 Department of Physics and Astronomy, University of Missouri, Columbia, MO 65211 USA; 4 York-Nanjing Joint Centre (YNJC) for spintronics and nano engineering, Department of Electronics, The University of York, York, YO10 3DD United Kingdom; 5 National Laboratory of Solid State Microstructures, College of Engineering and Applied Sciences, and Collaborative Innovation Center of Advanced Microstructures, Nanjing University, Nanjing, 210093 China

## Abstract

We have studied the Co_2_FeAl thin films with different thicknesses epitaxially grown on GaAs (001) by molecular beam epitaxy. The magnetic properties and spin polarization of the films were investigated by *in-situ* magneto-optic Kerr effect (MOKE) measurement and spin-resolved angle-resolved photoemission spectroscopy (spin-ARPES) at 300 K, respectively. High spin polarization of 58% (±7%) was observed for the film with thickness of 21 unit cells (uc), for the first time. However, when the thickness decreases to 2.5 uc, the spin polarization falls to 29% (±2%) only. This change is also accompanied by a magnetic transition at 4 uc characterized by the MOKE intensity. Above it, the film’s magnetization reaches the bulk value of 1000 emu/cm^3^. Our findings set a lower limit on the thickness of Co_2_FeAl films, which possesses both high spin polarization and large magnetization.

## Introduction

Spintronic devices rely on thin layers of magnetic materials, for they are designed to control both the charge and the spin current of the electrons. Half-metallic ferromagnets (HMFs) have only one spin channel for conduction at the Fermi level, while they have a band gap in the other spin channel^1–4^. Therefore, in principle this kind of material has 100% spin polarization for transport, which is perfect for spin injection, spin filtering, and spin transfer torque devices^5^. Among different types of HMFs, Co-based full Heusler alloys have attracted most interests due to their relatively high Curie temperature and low Gilbert damping coefficient^6–9^.

Although the future of applications is brilliant, most studies of Co_2_FeAl films are focused on relatively thicker films of more than 10 nm^5,10,11^. However, the ultra-thin film is essential for the miniaturization of electronic devices in some aspects. Magnetic tunnel junctions (MTJs) based on Co_2_FeAl_0.5_Si_0.5_^12^ and La_0.7_Sr_0.3_MnO_3_^13^ have been reported to achieve tunnel magnetoresistance (TMR) ratio of 386% at 300 K and transport spin polarization of 99.6% at 10 K, respectively. However, the device independent investigations on the spin polarization of half metals are limited, and mostly focused on Co_2_MnSi^6,14^. Therefore, in this manuscript, we have explored the magnetic properties and the spin-polarization of the ultra-thin Co_2_FeAl films ranging from 1 unit cell (uc) to 35 uc by *in-situ* magneto-optic Kerr effect (MOKE) and spin-resolved angle-resolved photoemission spectroscopy (spin-ARPES)^15^ at 300 K.

We have found that the films possess a combination of uniaxial and cubic anisotropy. The magnetization of all the films demonstrates a linear relationship with the thickness and a kink at 4 uc, above which it reaches the bulk value of 1000 emu/cm^3^ (Fig. S2)^16^. This suggests that a bulk magnetization is achieved for Co_2_FeAl thin films with a thickness of at least 4 uc. At greater thicknesses, we find a weak thickness dependence of the surface spin polarization at room temperature, which reaches 58% (±7%) for a 21-uc-thick film.

## Results

### High-quality Co_2_FeAl ultra-thin films

**Figure 1:**
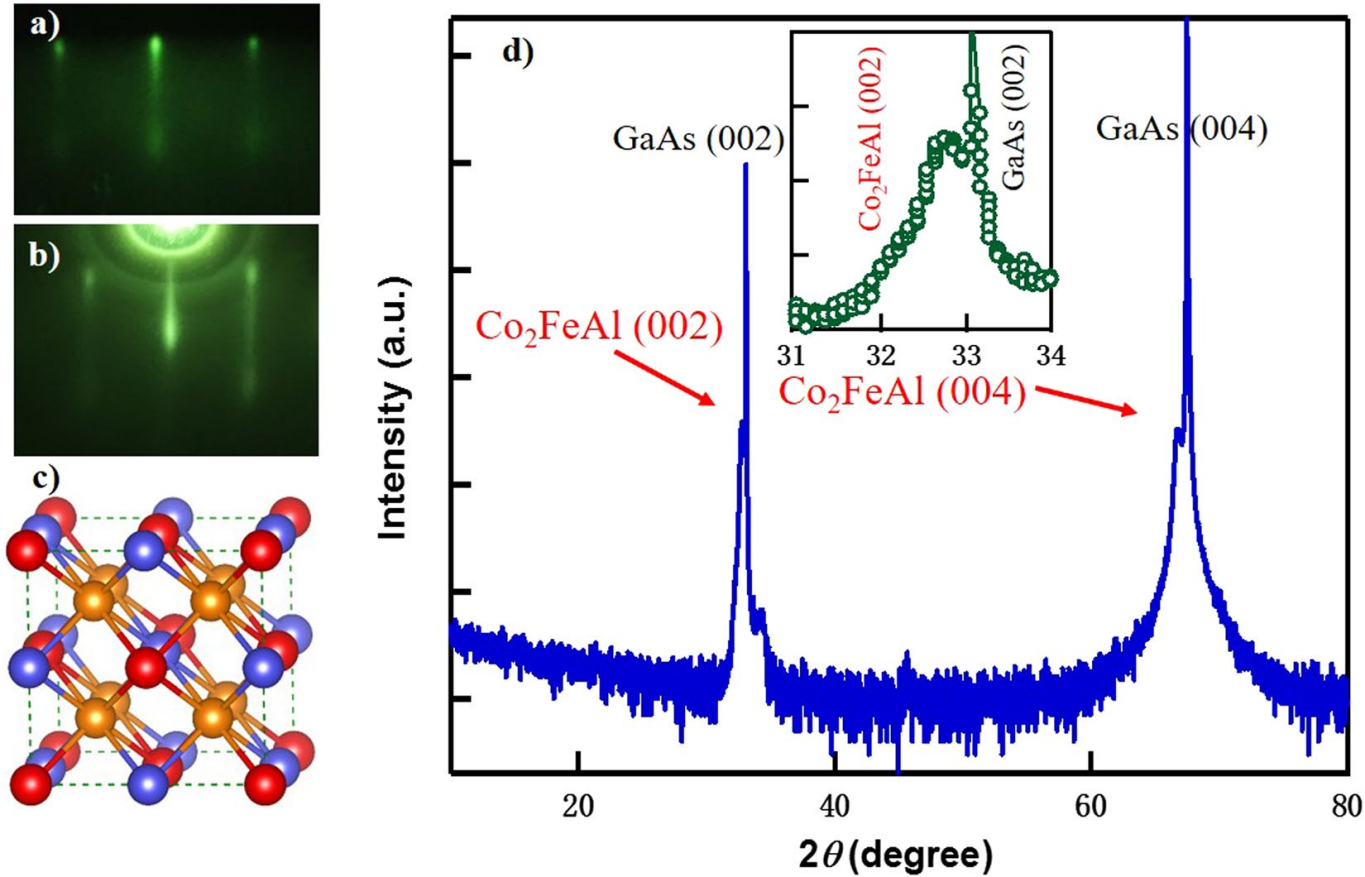
RHEED, structure model and XRD measurements of the Co_2_FeAl films. (**a**) and (**b**) RHEED patterns of a Co_2_FeAl (001) film with the electron beam along [110] & [100], respectively. (**c**) Schematic representation of Co_2_FeAl with L2_1_ structure. The golden, purple and red balls denote Co, Al and Fe atoms, respectively. (**d**) X-ray 2*q-w* diffraction patterns of a 13-uc-thick Co_2_FeAl film grown on GaAs (001) substrate. Top inset is a close view of the Co_2_FeAl (002) peak.

The Co_2_FeAl films were grown on GaAs (001) by molecular beam epitaxy (MBE). Real-time reflection high energy electron diffraction (RHEED) was used to monitor the *in-situ* growth dynamics with the electron beam along the [110] and [100] directions. Figure 1a,b present the RHEED patterns of an as-grown Co_2_FeAl film with a thickness of 20 uc. The sharp streaky lines indicate a flat surface morphology, thus the growth is smoothly pseudomorphic. The epitaxial relationship is Co_2_FeAl (001)[110] // GaAs (001)[110]. Figure 1d exhibits X-ray 2*θ*–*ω* diffraction pattern of a Co_2_FeAl film with a thickness of 13 uc. Though the film is ultra-thin, Co_2_FeAl (200) and (400) peaks can still be clearly observed, in addition to the peaks of GaAs substrate. From the diffraction peaks, the lattice constant of the film can be estimated as 5.70 Å, slightly smaller than the theoretical value of 5.727 Å. This suggests that our film is still under compressive strain induced by the GaAs substrate.

In theory, a perfect chemically and structurally ordered Co_2_FeAl crystal is L2_1_ phase. As exhibited in Fig. 1c, Co atoms (golden balls) sit at the eight vertexes of the cubic, while Fe (red balls) and Al atoms (purple balls) occupy the body center place alternately. It would be characterized by the peaks of superlattice reflections like (111) and (311)^17,18^. As the Fe and Al atoms mix with each other, B2 phase appears. In our case, the presence of both (200) and (400) peak indicates that our Co_2_FeAl films are in the B2 phase^19^. Besides the two main peaks, the absence of extra peaks suggests that our films possess a single crystal structure.

### *In-situ* longitudinal MOKE measurements

**Figure 2:**
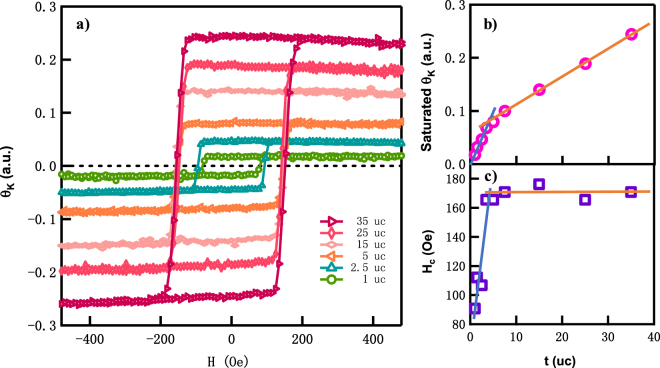
Thickness-dependent *in-situ* MOKE measurements of the Co_2_FeAl films. (**a**) In-plane magnetization measured at 300 K along $[\bar{1}\,1\,0]$ (easy axis direction) by a longitudinal-mode MOKE set up. Both thickness dependent (**b**) Kerr rotation and (**c**) coercive field estimated from (**a**) show a turning point at ~4 uc.

The magnetic properties of the Co_2_FeAl films were probed *in-situ* by MOKE measurements at room temperature. We have measured the longitudinal MOKE along $[\bar{1}\,0\,0]$, $[\bar{1}\,1\,0]$, [0 1 0] & [1 1 0] crystal orientations. It is interesting to notice that the magnetization of our Co_2_FeAl films exhibit a combination of uniaxial and cubic anisotropy. As the film thickness decreases, the uniaxial anisotropy becomes more pronounced with the easy axis along $[\bar{1}\,1\,0]$ direction and hard axis at [1 1 0] (Fig. S1). This uniaxial anisotropy may be induced by the Co_2_FeAl/GaAs interface, as the dangling bonds at the GaAs surface are all along $[\bar{1}\,1\,0]$. Along the easy axis, the field dependent MOKE signals (*θ*_*K*_) of various thicknesses are presented in Fig. 2a. Square hysteresis loops can be observed with thickness down to 1 uc. This strong remanence for 1 uc suggests that the long range ordering has formed between the Co and Fe nanoclusters through the ferromagnetic double exchange couplings^20^ at the early growth stage. As the film thickness increases, both *θ*_*K*_ and the coercivity increases. The coercivity saturates after the film thickness is beyond 4 uc.

The thickness dependent saturated Kerr rotation and coercive field extracted from Fig. 2a are presented in Fig. 2b,c, respectively. When the thickness increases from 0 to 4 uc, the saturated Kerr rotation increases linearly. And more interestingly, it passes through zero (blue solid line in Fig. 2b), suggesting that there are no magnetic dead layers, and the entire Co_2_FeAl film is ferromagnetic at room temperature. At the same time, the coercivity also increases linearly with the thicknesses, as indicated by the blue solid line in Fig. 2c. For thicker films (*t* > 4 uc), the Kerr rotation is also linearly dependent on the film thicknesses, with a smaller slope as fitted by the orange solid line in Fig. 2b. This is because the film thickness is still thinner than the detection depth of MOKE measurement, which is usually 10~50 nm^16,21^. Thus, the thicker the film is, the stronger the MOKE signal is. On the other hand, the coercivities stay constant (Fig. 2c), which are equal to the bulk value^22,23^. The magnetization and coercivity imply that the films thicker than 4 uc are bulk-like, while films thinner than 4 uc are mostly affected by the interface^16^.

### High spin polarization at the Fermi level

To investigate the spin polarization of the Co_2_FeAl films, samples were transferred under ultra-high vacuum to the ARPES chamber upon completing the film growth. This *in-situ* ARPES set-up prevents the contamination from ambient environment, thus it gives us a chance to observe the real spin polarization at the fresh Co_2_FeAl surface. Prior to the measurements, the magnetization direction was pulled to the easy axis along $[\bar{1}\,1\,0]$ direction, with an external magnetic field of 500 Oe. During the measurements, no out-of-plane spin polarization was observed, which confirms that the magnetization of our Co_2_FeAl films is in plane, as confirmed by our MOKE measurements.

**Figure 3:**
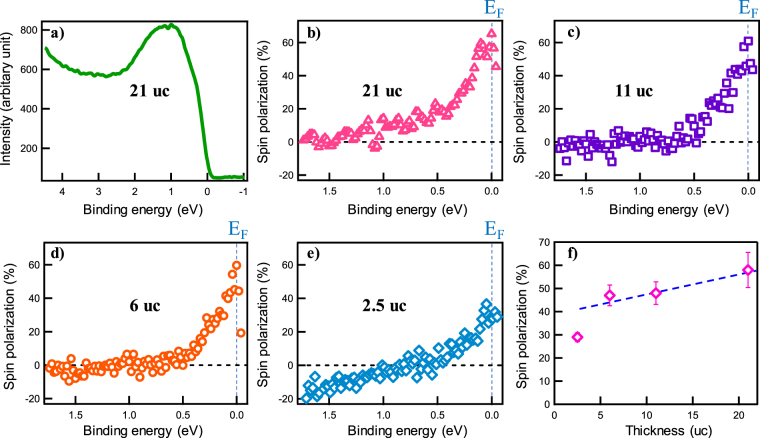
*In situ spin-resolved ARPES measurements*. (**a**) Spin-resolved photoemission spectra of the 21-uc-thick Co_2_FeAl film, probed by 21.2 eV photons at 300 K. (**b**–**e**) Spin polarizations of Co_2_FeAl films with thicknesses of 21 uc, 11 uc, 6 uc and 2.5 uc, respectively. (**f**) Spin polarizations at the Fermi level versus film thicknesses. It decreases slowly with film thicknesses decreasing from 21 uc to 6 uc, and then suddenly drops to 29% at 2.5 uc.

Figure 3 exhibits the representative spin-resolved photoemission spectra and the corresponding spin polarization at room temperature. A broad peak at ~1.0 eV in Fig. 3a comes from the combination of Co and Fe’s 3d electronic states, which is similar to the spectra of Co_2_MnSi films obtained in previous report^21^. The polarization of a free-electron beam can be determined by a spin-sensitive technique that involves scattering measurements from metals with strong spin orbit coupling^14^. Thus the spin polarization can be defined as:

1 $$p=\frac{\frac{{I}^{+}-{I}^{-}}{{I}^{+}+{I}^{-}}}{{S}_{eff}}$$

*I*^+^ and *I*^-^ represent the intensity spectra for majority and minority spins, respectively. *S*_*eff*_ means the Sherman function, representing the analyzing power or spin sensitivity of the polarimeter, which is equal to 0.16 ± 0.01 in our case^14,24^.

The magnitude of spin polarization of ferromagnetic materials is a key property for their application in spintronic devices, especially at room temperature. As shown in Fig. 3b–e, the spin polarization of Co_2_FeAl films exhibits a peak at the Fermi energy (E_F_), then decreases slowly with the binding energy increasing, and reaches zero beyond 1 eV. For the film of 2.5 uc, the peak value is much lower than the thicker films, and also the spin polarization goes to a negative value at higher binding energies, suggesting the swap of spin direction of the majority and the minority band. From the theoretical calculation^25^, the Fermi level crosses the majority band, touches the top of minority valence band, indicating the highest spin polarization at the Fermi level, which is in good agreement with our experimental observation. The thickness dependent spin polarization at the Fermi surface is exhibited in Fig. 3f. We find that the spin polarization decreases slowly as the film thickness is reduced from 21 uc to 6 uc, and drops to 29% (±2%) when the film thickness is reduced to 2.5 uc.

## Discussion

The spin polarization of 58% (±7%) for the Co_2_FeAl films with thickness of 21 uc is the highest value detected directly up to now for this materials system. However, it is still smaller than the expected 100% for half-metallic ferromagnets^21^. The plausible reasons may be local atomic disorder of Fe and Al atoms as demonstrated by XRD measurements or nonstoichiometric phase at the surface^26,27^. We have to point out that the measured spin polarization is at the interface between the Co_2_FeAl bulk and the vacuum, which may not be equal to the spin polarization at the interface between the Co_2_FeAl and MgO in a real TMR device. That interface could alter the spin polarization dramatically, as we can see in Fig. 3f. With film thickness decreasing to 2.5 uc, the strong attenuation of the spin polarization happens at E_F_, which may be due to the interface bonding or the site disorder, resulting in a spin polarization that is much less than the bulk.

In conclusion, we have grown single crystalline Co_2_FeAl films with B2 structure by MBE. The films exhibit a combination of uniaxial and cubic anisotropy. As the first direct observation of spin polarization for the Co_2_FeAl system, a high spin polarization of 58% (±7%) at the Fermi edge at room temperature was obtained by *in-situ* spin-resolved ARPES for a 21 uc-thick Co_2_FeAl film. *In-situ* MOKE measurements indicate that the thickness of the Co_2_FeAl film must reach at least 4 uc to achieve both a bulk magnetization and a high surface spin polarization with only a weak thickness dependence. Our work paves the way for the design and application of spintronic devices based on Co_2_FeAl films.

## Methods

### Epitaxial growth

To prepare the samples, we have used highly insulating GaAs (001) epi-ready wafers whose lattice constant is very close to Co_2_FeAl (001)^28^, and the Co_2_FeAl thin films were grown in an ultra-high vacuum MBE system with the base pressure below 3 × 10 ^−9^ mbar. Before the growth, GaAs (001) substrates were annealed at 580 °C to remove Gallium oxide. We used two e-beam evaporators for Co and Fe and a Knudsen cell for Al with the substrate sitting at 250 °C. The deposition rate of ~1 uc per min was measured by a quartz microbalance, which was calibrated by thickness measurements using atomic force microscopy (AFM).

### Structural characterization

The crystal structure was examined by a high resolution single crystal X-ray diffractometer (Bruker D8 Discover). The incident X-ray is from Cu-Kα emission and has a wavelength of 1.5418 Å. The scan mode is *θ*-2*θ*.

### *In-situ* MOKE Characterization

The MOKE loops were collected during growth in the longitudinal geometry using an electromagnet with a maximum field of 500 Oe, and an intensity stabilized HeNe laser (633 nm) at 300 K. The MOKE signal is proportional to the Kerr effect, the angle between the polarizer and the analyzer, and the intensity of the light. During the *in-situ* MOKE measurement, cares were taken not to move any optical components in order to keep the laser intensity constant^16,21^.

### Spin-arpes measurements

*In-situ* Spin-ARPES measurements were performed using a lab-based Spin-ARPES system consisting of a SPECS PHOIBOS 150 hemisphere analyzer with 3D Micro-Mott detector and UVS 300 helium lamp (21.2 eV). The 3D Micro-Mott detector is equipped with 4 channels which allows us to measure both in-plane and out-of-plane spin components with an energy resolution of 150 meV at room temperature. We operated the Mott detector at a scattering energy of 25 keV, and an inelastic energy window equal to 800 eV, which leads to a Sherman function of 0.16 ± 0.01. The spectrometer was fixed at a large acceptance angle (±15°), which covered the complete Brillouin zone. The base pressure in Spin-ARPES chamber is better than 3 × 10^−10^ mbar, and the samples were measured at 300 K.

## Electronic supplementary material

Supporting Information
