# Supplementary material for: Direct observation of high spin polarization in Co2FeAl thin films
Source: Sci Rep. 2018 May 23;8:8074. doi: 10.1038/s41598-018-26285-9 (PMC5966392; doi:10.1038/s41598-018-26285-9)
Supplement: Supplementary file 1 — Supporting Information [file 41598_2018_26285_MOESM1_ESM.pdf]

Supporting Information

Direct observation of high spin polarization in Co_2FeAl thin films

Xiaoqian Zhang,[1] Huanfeng Xu,[1] Bolin Lai,[1] Qiangsheng Lu,[2,3] Xianyang Lu,[4] Yequan Chen,[1] Wei Niu,[1] Chenyi Gu,[5] Wenqing Liu,[1,4] Xuefeng Wang,[1] Chang Liu,[2] Yuefeng Nie,[5] Liang He,[1] Yongbing Xu[1,4*]*

[1]Jiangsu Provincial Key Laboratory of Advanced Photonic and Electronic Materials, Collaborative Innovation Center of Advanced Microstructures, School of Electronic Science and Engineering, Nanjing University, Nanjing 210093, China

[2]Department of Physics, Southern University of Science and Technology, Shenzhen, Guangdong, 518055, China

[3]Department of Physics and Astronomy, University of Missouri, Columbia, MO, 65211, USA

[4]York-Nanjing Joint Centre (YNJC) for spintronics and nano engineering, Department of Electronics, The University of York, YO10 3DD, United Kingdom

[5]National Laboratory of Solid State Microstructures, College of Engineering and Applied Sciences, and Collaborative Innovation Center of Advanced Microstructures, Nanjing University, Nanjing 210093, China

Corresponding Authors

* L.H: heliang@nju.edu.cn

* Y.B.X: ybxu@nju.edu.cn

SUPPORTING INFORMATION

S1. *In-situ* longitudinal MOKE measurement of 5-uc-thick Co_2FeAl film

The magnetic properties of the Co_2FeAl films were probed *in-situ* by MOKE measurements at room temperature. As shown in Fig. S1, longitudinal Kerr rotation was measured at 300 K along [$\bar{1}$ 0 0], [$\bar{1}$ 1 0], [0 1 0] & [1 1 0], respectively. The red (blue) lines represent that the magnetic field goes from negative (positive) to positive (negative). The 5-uc-thick Co_2FeAl film exhibits in-plane uniaxial magnetic anisotropy, which is related to the interfacial bonding between As and Co or Fe atoms[1].

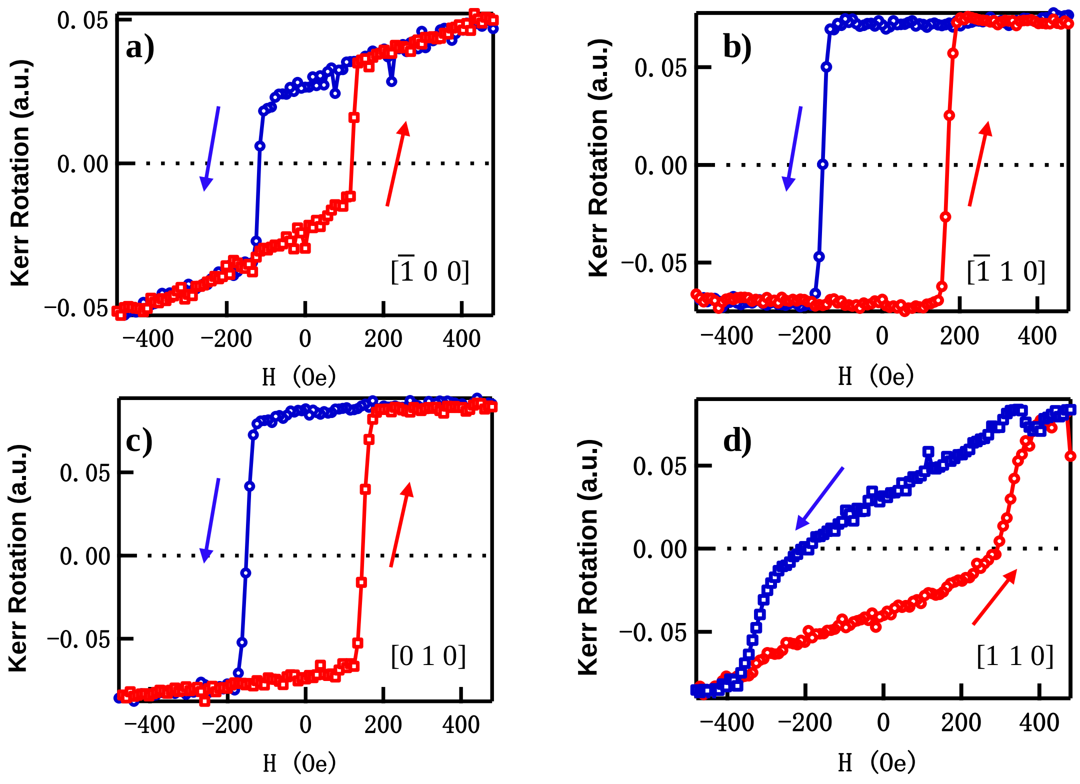

FIG. S1 In-situ longitudinal MOKE measurement of 5-uc-thick Co2FeAl film. (a)-(d) Longitudinal Kerr rotation measured at 300 K along [$\bar{1}$ 0 0], [$\bar{1}$ 1 0] (easy axis direction), [0 1 0] & [1 1 0], respectively. The red (blue) lines represent that the magnetic field goes

from negative (positive) to positive (negative). The 5-uc-thick Co2FeAl film exhibits in-plane uniaxial magnetic anisotropy.

S2. VSM measurement of 35-uc-thick Co_2FeAl film.

Upon the growth was finished, sample was capped with 2-nm Al and then transferred out of chamber. VSM measurement of 35-uc-thick Co_2FeAl film was carried out at 300 K along $[\bar{1}\ 0\ 0]$, $[\bar{1}\ 1\ 0]$ (easy axis direction), [0 1 0] & [1 1 0], respectively. The film's magnetization reaches the bulk value of 1000 emu/cm^3.

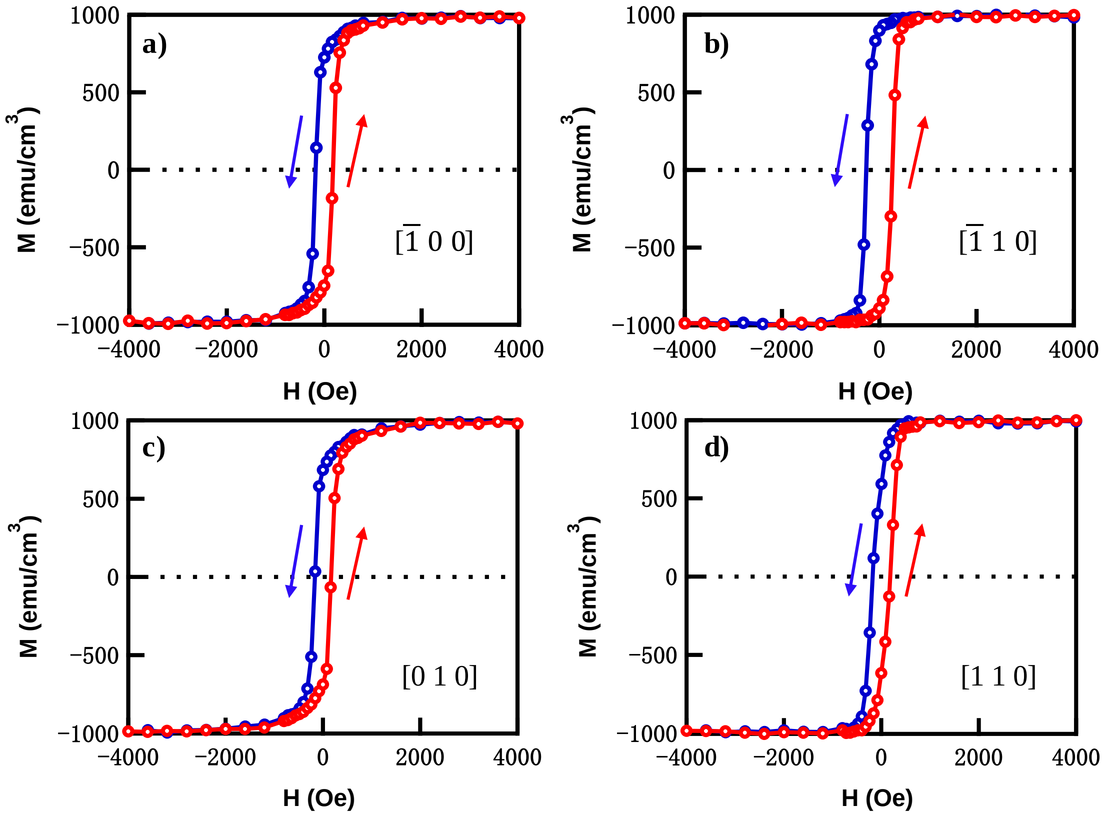

FIG. S2 **VSM measurement of 35-uc-thick Co_2FeAl film.** (a)-(d) Magnetization of 35-uc-thick Co_2FeAl film measured at 300 K along $[\bar{1}\ 0\ 0]$, $[\bar{1}\ 1\ 0]$ (easy axis direction), [0 1

0] & [1 1 0], respectively. The red (blue) lines represent that the magnetic field goes from negative (positive) to positive (negative).

S3. EDC and spin polarization.

Fig. S3 exhibits the representative spin-resolved photoemission spectra and corresponding spin polarization of Co_2FeAl films ranging from 21 uc to 2.5 uc measured at room temperature. Then we can get the thickness dependent spin polarization[2-4].

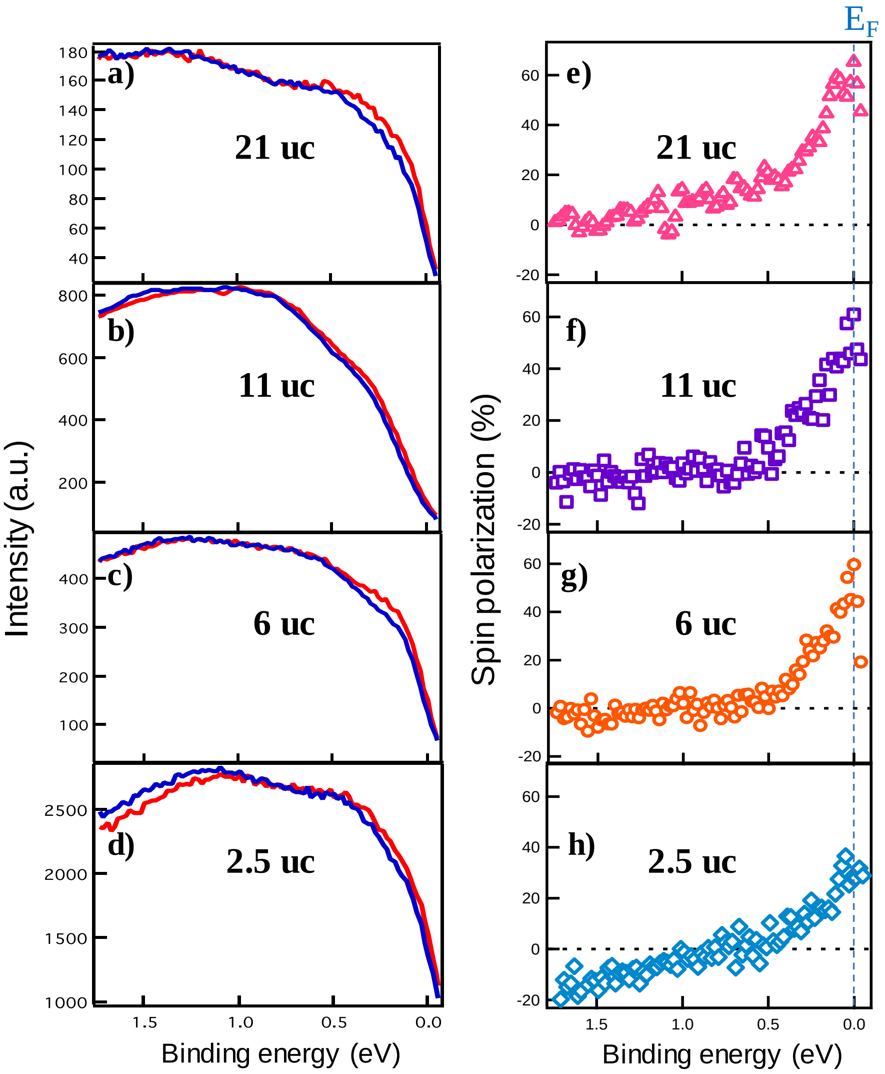

FIG. S3 **EDC and spin polarization.** Spin-resolved photoemission spectra (a-d) and spin polarization (e-h) of Co_2FeAl films, detected at 21.2 eV photon energy. Red (blue) lines denote spin up (down) spectra obtained by Mott detector.

References

1 Xu, Y. B. *et al.* Evolution of the ferromagnetic phase of ultrathin Fe films grown on GaAs(100)-4x6. *Physical Review B* **58**, 890-896, (1998).

2 Jourdan, M. *et al.* Direct observation of half-metallicity in the Heusler compound Co_2MnSi. *Nature communications* **5**, 3974, (2014).

3 Kurt, H., Rode, K., Venkatesan, M., Stamenov, P. & Coey, J. M. D. High spin polarization in epitaxial films of ferrimagnetic Mn_3Ga. *Physical Review B* **83**, 020405(R) (2011).

4 Hahn, M., Schönhense, G., Jorge, E. A. & Jourdan, M. Significant spin polarization of Co_2MnGa Heusler thin films on MgO(100) measured by ultraviolet photoemission spectroscopy. *Appl Phys Lett* **98**, 232503, (2011).
